# EHMTI-0348. Refractive errors in patients with migraine headache

**DOI:** 10.1186/1129-2377-15-S1-M6

**Published:** 2014-09-18

**Authors:** A Gunes, S Demirci, L Tok, O Tok, HR Koyuncuoglu, VA Yurekli

**Affiliations:** 1 Department of Ophthalmolgy, Süleyman Demirel University, Isparta, Turkey; 2 Department of Neurology, Süleyman Demirel University, Isparta, Turkey; 3 Department of Ophthalmology, Süleyman Demirel University, Isparta, Turkey

## Introduction

Migraine is one of the most common debilitating diseases. Despite of intensive research in the pathogenesis and treatment of migraine, its relationship between refractive error have been controversial.

## Aims

To evaluate refractive errors in patients with migraine headache and to compare with healthy subjects.

## Methods

This prospective case-control study includes patients with migraine and age- and sex- matched healthy subjects. Clinical and demographic characteristics of the patients were noted. Then detailed ophthalmological examination were performed containing spherical refractive error, astigmatic refractive error, spherical equivalent (SE), anisometropia, best corrected visual acuity, intraocular pressure, slit lamp biomicroscopy, fundus examination, axial length, anterior chamber depth, and central corneal thickness. Spectacle use in migraine and control groups was compared. Also, the relationship between refractive components and migraine headache variables were investigated.

## Results

Seventy-seven migraine patients with mean age of 33.27±8.84 years and 71 healthy subjects with mean age of 31.15±10.45 years were enrolled (p=0.18). The migraine patients had higher degrees of astigmatic refractive error, SE, and anisometropia when compared with the control subjects (p= 0.01, p= 0.03, p= 0.02, respectively).

## Conclusions

Migraine patients may have higher degrees of astigmatism, SE, and anisometropia. Therefore, they should have ophthalmological examination regularly to ensure that their refractive errors are appropriately corrected.

Seventy-seven migraine patients with mean age of 33.27±8.84 years and 71 healthy subjects with mean age of 31.15±10.45 years were enrolled (p=0.18). The migraine patients had higher degrees of astigmatic refractive error, SE, and anisometropiawhencompared with the control subjects (p= 0.01, p= 0.03, p= 0.02, respectively).

No conflict of interest.

